# Multilevel and quasi-Monte Carlo methods for uncertainty quantification in particle travel times through random heterogeneous porous media

**DOI:** 10.1098/rsos.170203

**Published:** 2017-08-09

**Authors:** D. Crevillén-García, H. Power

**Affiliations:** 1 School of Engineering, University of Warwick, Coventry CV4 7AL, UK; 2 Faculty of Engineering, University of Nottingham, Nottingham NG7 2RD, UK

**Keywords:** groundwater flow, partial differential equations with random coefficients, uncertainty quantification, quasi-Monte Carlo, multilevel methods

## Abstract

In this study, we apply four Monte Carlo simulation methods, namely, Monte Carlo, quasi-Monte Carlo, multilevel Monte Carlo and multilevel quasi-Monte Carlo to the problem of uncertainty quantification in the estimation of the average travel time during the transport of particles through random heterogeneous porous media. We apply the four methodologies to a model problem where the only input parameter, the hydraulic conductivity, is modelled as a log-Gaussian random field by using direct Karhunen–Loéve decompositions. The random terms in such expansions represent the coefficients in the equations. Numerical calculations demonstrating the effectiveness of each of the methods are presented. A comparison of the computational cost incurred by each of the methods for three different tolerances is provided. The accuracy of the approaches is quantified via the mean square error.

## 1. Introduction

The Monte Carlo (MC) method is a widely used and effective approach for uncertainty quantification (UQ) in systems of ordinary/partial differential equations (ODEs/PDEs) with random coefficients [1,2]. The implementation of this method is straightforward, it can be applied to any type of problem including nonlinear problems, it is possible to compute an estimate of the error as part of the solution process and it does not suffer from the so-called *curse of dimensionality*. In this method, the relevant parameter values are drawn from their probability distributions and the governing equations are solved for such samples. This gives a set of samples of the output variables, from which various statistics of the quantity of interest (QoI), such as the mean and the variance, can be calculated. The main constraint of this method is its slow rate of convergence: the error decreases approximately as the inverse of the square root of the number of samples [2].

In this paper, we investigate three existing methods for outperforming MC, namely, multilevel Monte Carlo (MLMC) [3], quasi-Monte Carlo (QMC) [4] and multilevel quasi-Monte Carlo (MLQMC) [5]. We apply these methodologies to the problem of travel time estimation in heterogeneous porous media. This is of central importance in a series of engineering applications ranging from groundwater management to groundwater remediation. It also involves the development of mathematical models for reactive transport in porous media. These models are used to assess, for instance, groundwater contamination, CO_2_ sequestration, residence time distributions, etc. The QoI considered in this study will be the result of an ODE (the transport equation (2.4)) which uses a solution of a PDE with random inputs (equation (2.2)). Multilevel methods have been proved [2] to reduce significantly the classical MC asymptotic computational cost during the UQ in groundwater flow models in porous media. These methods exploit the linearity of the expectation, by expressing the QoI of a given problem on the finest spatial grid of the computational domain in terms of the same quantity on a relatively coarser grid and correction terms. The dramatic reduction in cost associated with the MLMC method over standard MC is due to the fact that most of the uncertainty can be captured on the coarsest grids, and thus, the number of realizations needed on the finest grids is greatly reduced. The QMC method is based on quasi-random sequences, which are deterministic alternatives to pseudo-random sequences [6,7]. While pseudo-random sequences try to mimic the properties of random sequences, quasi-random sequences are designed to provide better uniformity than a random sequence and hence faster convergence for quadrature formulae [8]. In practical terms, QMC uses uniformly spaced generated inputs from previously sampled quasi-random sequences [8] to estimate the QoI, providing a better rate of convergence than MC, and consequently, reducing significantly the computational cost in an uncertainty analysis.

The outline of this paper is as follows. In §2, we present the governing equations for our physical problem, we show how to model the hydraulic conductivity as a log-Gaussian random field, and finally, we describe the numerical method used to solve the equations with random coefficients. In §3, we describe the four MC simulation methodologies in a general context and show the algorithms used for implementation. In §4, we present and discuss our numerical results for the application of the four MC methods to a two-dimensional model problem. In §5, we give our conclusions and make some suggestions for future work.

## 2. Mathematical model

The classical equations governing (steady-state) single-phase subsurface flow, subject to suitable boundary conditions, consist of Darcy’s Law coupled with an incompressibility condition [2,9,10]:

2.1 $$\text{q}+K\nabla h=\text{g },\;\nabla \cdot \text{q}=0,\;\mathrm{in}\;R\subset R^{2},$$

where *h* (m) denotes the pressure head, *K* (m s^−1^) the hydraulic conductivity, **q** (m^2^ s^−1^) the Darcy flux and **g** represents the source terms.

The process considered in this study is the flow of an incompressible liquid in a horizontal confined aquifer. For this problem, we consider a square flow domain $R=[0,1]\times [0,1]\subset R^{2}$, and the source terms are set to zero for simplicity, i.e. **g**≡0. The governing equations defined in (2.1) are coupled to yield a single equation for the pressure head:

2.2 ∇⋅(K(x)∇h(x))=0,x=(x,y)∈R.

The QoI to be considered in this problem is the travel time *τ* that a convected particle released at the centre of the domain R takes to reach the boundary of the domain, ∂R, i.e. from the point (*x*_0_,*y*_0_)=(1/2,1/2) to (1,y)∈∂R. The boundary conditions considered are

2.3 $$h(0,y)=250,\;h(1,y)=0,\;\frac{\partial h}{\partial y}(x,0)=0,\;\frac{\partial h}{\partial y}(x,1)=0.$$

To compute the travel time *τ*, we let **x**=***ζ***(*t*)=(*ζ*_1_(*t*),*ζ*_2_(*t*)) be the location of a particle. After the pressure is calculated from (2.2), the trajectory ***ζ***(*t*) is computed by solving the transport equation (2.4) subject to the initial condition $\zeta (0)=(\frac{1}{2},1/2)$. We then determine the time *τ* for which *ζ*_1_(*τ*)=1, i.e. the convected particle lies on the right boundary, by solving [11,12]

2.4 $$\frac{d\zeta (t)}{dt}=-\frac{K(\zeta )}{\phi }\nabla h(\zeta ),$$

where *ϕ* is the rock porosity (dimensionless), i.e. the ratio of void volume in a rock to total volume. To solve equation (2.2) in R, we used a numerical code based on the standard cell-centred finite-volume method. After the pressure field *h* is computed, for simplicity, the spatial gradient of heads is approximated by using the central finite difference (*h*_*i*+1,*j*_−*h*_*i*,*j*_)/|**x**_*i*+1,*j*_−**x**_*i*,*j*_|, where **x**_*i*,*j*_ denotes the centroid of each cell in the computational mesh (see [2] for full details). Equation (2.4) was solved by direct Euler integration.

In this application, the uncertain inputs for the simulator will be the values of the hydraulic conductivity *K* at each of the nodes of the computational domain. The simulator output will be the travel time. It is common in groundwater flow studies [13–16] to model *K* as a log-Gaussian random field, i.e. to replace the conductivity by a scalar valued field, *K*(**x**), whose log is Gaussian, *Z*(**x**):=log*K*(**x**) or *K*(**x**)=exp(*Z*(**x**)). By doing this, we also guarantee that *K*>0 in R. Several studies [17–19] have shown that although the conductivity values can exhibit large spatial variations, these are spatially correlated. A correlation function that has been extensively used [2,11,19,20] for modelling the correlation of *Z*(**x**) is the following exponential covariance function:

2.5 $$c({\text{x}}_{i},{\text{x}}_{j})=\sigma^{2}\exp\left(\frac{-∥{\text{x}}_{i}-{\text{x}}_{j}{∥}_{2}}{\lambda }\right)\;{\text{x}}_{i},{\text{x}}_{j}\in R,$$

where *λ* denotes the correlation length and *σ*^2^ is the process variance. In groundwater flow applications, the geostatistical/variogram parameters, in this case, *λ* and *σ*^2^, must be chosen according to the geostatistics of the considered porous medium. In this work, the parameters representing the conductivity fields have been selected from ranges gathered from the literature. Values around 0.3 for *λ* and around 1.0 for *σ*^2^ appear to be the preferred in similar studies (e.g. [14–16]). Thus, in this paper, we will use *λ*=0.3 and *σ*^2^=1.0. Note also that an appropriate discretization scheme for this type of models must be designed according to the value of *λ*; in other words, the size of the computational domain has to be chosen significantly larger than the value of *λ* and also allow *λ* to be large enough to be taken into account in the numerical formulation [2], i.e. in our case, larger than the distance between centroids in adjacent cells.

To generate samples of *K*(**x**) at the nodes of the computational domain, first, we need to generate samples of Gaussian field *Z*(**x**) at such nodes. One of the most popular methods to generate different (Gaussian distributed) *Z*(**x**) is the Karhunen–Loéve (KL) expansion method [2,13–16,21,22]. This method provides an approximation (due to the truncation of an infinite series) of the permeability fields at all the points of the continuous domain, which can be sampled afterwards on any grid. In order to avoid adding extra errors (arisen from the truncation of the KL expansion) to the model and produce more accurate representations of the hydraulic conductivity, alternative methods might be considered, for instance, the circulant embedding algorithm [23–25]. The circulant embedding method provides fast and exact representations of the Gaussian field but requires the use of the fast Fourier transform method, and thus, it is not straightforward to implement. Two alternatives to the circulant embedding method for producing exact decompositions of the covariance matrix associated to the correlation function given in (2.5) are the Cholesky method [11,25,26] and the KL decomposition [22,27]. These methods are not recommended for covariance functions that are differentiable at zero lag distance, e.g. the square exponential (or Gaussian) correlation function [22,28]. In those cases, the associated covariance matrix is likely to become extremely ill-conditioned [29,30]. They could be also inappropriate for problems in which the simulator necessitates an extremely fine discretization of the computational domain [30], but this does not apply to the problem considered in this paper. Conversely, the main advantages of this approach is that it only requires a single eigen-decomposition of the covariance matrix, the results of which are stored and used to generate new realizations of the permeability field very cheaply, and furthermore, its implementation is very simple and straightforward. In this paper, we will opt for the KL decomposition method, which is described briefly next (for full details, e.g. [22,25]).

Let ${\left\{{\text{x}}_{j}\right\}}_{j=1}^{M}\subset R$ be the set of nodes for a given discretization of the problem domain R. To generate samples of *Z*(**x**), we let **C** be the positive semi-definite covariance matrix associated to the function *c*, i.e. *C*_*ij*_=*c*(**x**_*i*_,**x**_*j*_), ${\text{x}}_{i},{\text{x}}_{j}\in R$. This matrix admits an eigen-decomposition [26]: $\text{C}=(\Phi \Lambda^{1/2}){\left(\Phi \Lambda^{1/2}\right)}^{⊺}$, where *Λ* is the *M*×*M* diagonal matrix of ordered decreasing eigenvalues *λ*_1_≥*λ*_2_≥⋯≥*λ*_*M*_≥0, and *Φ* is the *M*×*M* matrix whose columns ***ϕ***_*i*_, *i*=1,…,*M*, are the eigenvectors of *C*. Let $\xi_{i}∼N(0,1)$, *i*=1,…,*M*, be independent and identically distributed (i.i.d.) random variables. We can draw samples from Z∼N(m,C) at the points **x**_*j*_ using the KL decomposition of $\text{Z}:={\left(Z_{1},\ldots ,Z_{M}\right)}^{⊺}$ using the following [25]:

2.6 $${\left(Z_{1},\ldots ,Z_{M}\right)}^{⊺}=\text{m}+\Phi \Lambda^{1/2}{\left(\xi_{1},\ldots ,\xi_{M}\right)}^{⊺}.$$

Without loss of generality, we will set **m**≡**0** in (2.6), and thus, the discrete random permeability field is therefore given by

2.7 $$\text{K}={\left(\exp(Z_{1}),\ldots ,\exp(Z_{M})\right)}^{⊺}.$$

The terms $\xi_{i}∼N(0,1)$ above will be called *KL coefficients*. Now, for each new ensemble $\left\{\xi_{1}^{j},\ldots ,\xi_{M}^{j}\right\}$, j∈N, of random variables $\xi_{i}^{j}∼N(0,1)$, we can generate a new realization of the conductivity ${\text{K}}^{j}\in R^{M}$. Note that this method only provides values of the conductivity at the nodes and not in the whole continuum R.

In the following section, we include a review of the literature related to the implementation of the four MC methods.

## 3. Monte Carlo simulation methods

Let (Ω,F,P) be a probability space. Let ${\text{X}}_{M}:={\left(\xi_{1},\ldots ,\xi_{M}\right)}^{⊺}$ be the random vector formed with a given ensemble of *M* KL coefficients which yields to a discrete random permeability field $\text{K}\in R^{M}$. Let $T_{M}=f({\text{X}}_{M})\in R$, where *f* denotes the travel time simulator, be the approximation of the travel time obtained with the simulator based on a computational domain of *M* nodes ${\left\{{\text{x}}_{j}\right\}}_{j=1}^{M}$. We denote by *T* the *true* (underlying) travel time random variable T:Ω→R solution of (2.2), and assume that the expected value $E[T_{M}]\to E[T]$, as M→∞, and that (in mean) the order of convergence is *α*>0 (see [2,3] for further details), i.e.

3.1 $$|E[T_{M}-T]|\leq C_{\alpha }M^{-\alpha }$$

for some constant *C*_*α*_. We are interested in estimating E[T]. Thus, given M∈N sufficiently large, we compute approximations (or estimators) ${\hat{T}}_{M}$ of $E[T_{M}]$ and quantify the accuracy of our approximations via the root mean square error (RMSE):

3.2 $$e({\hat{T}}_{M}):={\left(E[{\left({\hat{T}}_{M}-E[T]\right)}^{2}]\right)}^{1/2}.$$

We will denote by $C_{\varepsilon }$ the computational *ε*-Cost used to achieve an RMSE $e({\hat{T}}_{M})\leq \varepsilon$. This *ε*-Cost is quantified by the number of floating point operations that are needed to achieve an RMSE of $e({\hat{T}}_{M})\leq \varepsilon$.

**Figure 1.**
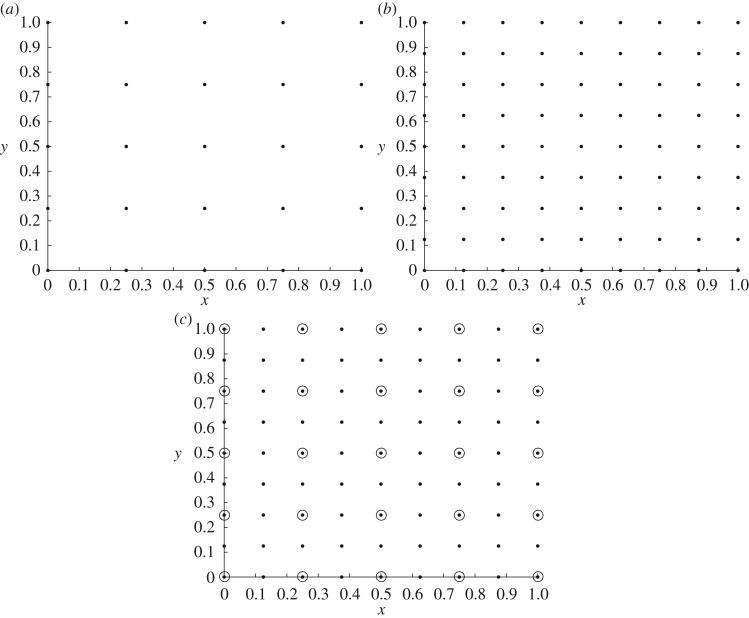
(*a*) Example of a grid of 25 nodes and (*b*) a grid of 81 nodes. (*c*) Grid of 25 nodes (circles) seen as a subgrid of a grid of 81 nodes (dots) for the domain *D*=[0,1]×[0,1].

As it could be well known to the reader (but it is important to remark here before discussing the MLMC method), when solving a system of PDEs, a computer model needs to retain all the important features of the physical domain (a continuum medium) of the problem and reduce them into a simplified form, called the computational domain (a discrete set of points). Throughout this paper, we will use the term *grid* for the structured distribution of points, called *nodes*, that form the computational domain used by the computer model to solve the equations, and *M* will denote the number of nodes which form the corresponding grid. According to this, given two grids *M*_*i*_ and *M*_*j*_ with i<j, i,j∈N, we will say that *M*_*i*_ is a *subgrid* of *M*_*j*_, and we will write *M*_*i*_<*M*_*j*_, if all the nodes contained in *M*_*i*_ are also contained in *M*_*j*_. We will then say that *M*_*i*_ is *coarser* than *M*_*j*_ and conversely that *M*_*j*_ is *finer* than *M*_*i*_. For solving efficiently a system of PDEs, choosing *M* sufficiently large corresponds to choosing a fine enough grid that guarantees that the computer model is providing an accurate approximation of the true solution of the problem. Figure 1 shows an example of two grids for the same physical domain used by a computer model.

In the following sections, we describe how to implement each of the MC methods. Note that while for all of the methods the QoI is $E(T_{M}$), in each of the methods we use a different estimator to approximate $E(T_{M})$.

### 3.1. Classical Monte Carlo simulation method

We define the standard MC estimator for estimating $E(T_{M})$ as follows:

3.3 $${\hat{T}}_{M,N}^{\mathrm{MC}}:=\frac{1}{N}\sum_{i=1}^{N}T_{M}^{\left(i\right)},$$

where $T_{M}^{\left(i\right)}$ is the *i*th sample of *T*_*M*_ and *N* independent samples are computed in total. Note that $E[{\hat{T}}_{M,N}^{\mathrm{MC}}]=E[T_{M}]$, i.e. ${\hat{T}}_{M,N}^{\mathrm{MC}}$ is an unbiased estimator of $E[T_{M}]$. We assume that the cost to compute one sample $T_{M}^{\left(i\right)}$ of *T*_*M*_ is

3.4 $$C(T_{M}^{\left(i\right)})\leq M^{\gamma },\;\text{for some}\;\gamma >0$$

and hence the total cost of the MC estimator satisfies [2]

3.5 $$C({\hat{T}}_{M,N}^{\mathrm{MC}})\leq NM^{\gamma }.$$

The MSE of ${\hat{T}}_{M,N}^{\mathrm{MC}}$ can be expressed as follows [2]:

3.6 $$e{\left({\hat{T}}_{M,N}^{\mathrm{MC}}\right)}^{2}=\frac{V[T_{M}]}{N}+{\left(E[T_{M}-T]\right)}^{2},$$

where $V[T_{M}]$ is the variance of the MC estimator, which represents the sampling error and decays inversely with the number of samples. The second term on the right-hand side is the square of the error in mean between *T*_*M*_ and *T*, also called the discretization error or the *bias*. Thus, once we have obtained a sufficient resolution of the problem by choosing a fine enough grid for the domain R (i.e. *M* large), the condition to achieve an accurate approximation of our QoI E[T] lies in generating a large number of samples *N* [3]. To bound the RMSE by *ε*, we can seek to bound each term in equation (3.6) by *ε*^2^/2. Note that, for the second term, it is sufficient to choose $M=M_{L}\geq {\left(\varepsilon /(\sqrt{2}C_{\alpha })\right)}^{-1/\alpha }$.

### 3.2. Multilevel Monte Carlo simulation

The main idea behind the MLMC simulation method is to start obtaining approximations of *T* from several grids, starting by the coarsest and stopping when the given MSE has been numerically achieved. For a detailed description of the method, the reader is referred to [2,3]. In this section, we only give a brief summary of the practicality of the approach.

Let {*M*_ℓ_:ℓ=0,…,*L*} be an increasing sequence of embedded grids in N called *levels*, i.e. *M*_0_<*M*_1_<⋯<*M*_*L*_=:*M*. The goal is to avoid estimating $E[T_{M_{\ell }}]$ from a very fine level ℓ, but instead to estimate the correction with respect to the next lower level, i.e. $E[Y_{\ell }]$, where *Y*_ℓ_:=*T*_*M*_ℓ__−*T*_*M*_ℓ−1__. Setting for simplicity *Y*_0_:=*T*_*M*_0__ and using the linearity of the expectation operator, we have

3.7 $$E[T_{M}]=E[T_{M_{0}}]+\sum_{\ell =1}^{L}E[T_{M_{\ell }}-T_{M_{\ell -1}}]=\sum_{\ell =0}^{L}E[Y_{\ell }].$$

All the terms *E*[*Y*_ℓ_] in the sum are independent and thus we estimate each of the expectations individually. Let ${\hat{Y}}_{\ell }$ be an unbiased estimator for $E[Y_{\ell }]$, in this case, the standard MC estimator with *N*_ℓ_ samples:

3.8 $${\hat{Y}}_{\ell ,N_{\ell }}^{\mathrm{MC}}:=\frac{1}{N_{\ell }}\sum_{i=1}^{N_{\ell }}(T_{M_{\ell }}^{\left(i\right)}-T_{M_{\ell -1}}^{\left(i\right)}),$$

then the multilevel estimator is defined as

3.9 $${\hat{T}}_{M}^{\mathrm{ML}}:=\sum_{\ell =0}^{L}{\hat{Y}}_{\ell }.$$

We will denote the MLMC estimator by ${\hat{T}}_{M,\{N_{\ell }\}}^{\mathrm{MLMC}}$, where the individual terms are estimated using the standard MC, ${\hat{Y}}_{\ell ,N_{\ell }}^{\mathrm{MC}}$.

**Figure 2.**
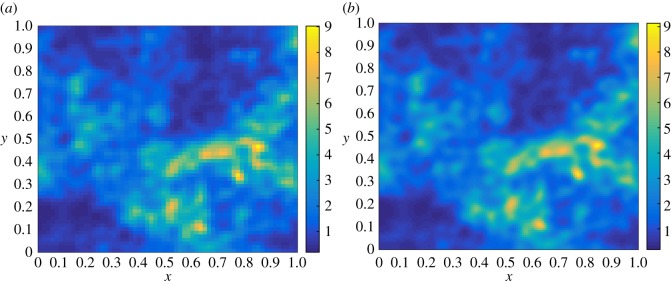
Two samples of the same random permeability field in two consecutive levels ℓ (*a*) and ℓ+1 (*b*) to be used as input in the MLMC method. In this example, we used ℓ=3.

Note that, for computing the quantities $T_{M_{\ell }}^{\left(i\right)}-T_{M_{\ell -1}}^{\left(i\right)}$ for ℓ=1,…,*L*, the terms $T_{M_{\ell }}^{\left(i\right)}$ and $T_{M_{\ell -1}}^{\left(i\right)}$ are simulated separately, each of them from the same random sample *ω*^(*i*)^∈*Ω* restricted to the corresponding level ℓ, i.e. we use a coarsened version of the same input used for $T_{M_{\ell }}^{\left(i\right)}$ in calculating $T_{M_{\ell -1}}^{\left(i\right)}$ (see figure 2 for clarification). As all the expectations $E[{\hat{Y}}_{\ell }]$ are estimated independently, the variance of the MLMC estimator is $V[{\hat{T}}_{M}^{\mathrm{ML}}]=\sum_{\ell =0}^{L}N_{\ell }^{-1}V[Y_{\ell }]$, and so the MSE is

3.10 $$e{\left({\hat{T}}_{M}^{\mathrm{ML}}\right)}^{2}:=E[{\left({\hat{T}}_{M}^{\mathrm{ML}}-E[T]\right)}^{2}]=\sum_{\ell =0}^{L}\frac{V[Y_{\ell }]}{N_{\ell }}+{\left(E[T_{M}-T]\right)}^{2}.$$

We see that the MSE for the multilevel estimator consists of two terms, the variance of the estimator and the approximation error. To bound the RMSE by *ε*, we can seek to bound each term above by *ε*^2^/2. Note that the second term is exactly the same as in equation (3.6) and so it is sufficient to choose $M=M_{L}\geq {\left(\varepsilon /(\sqrt{2}C_{\alpha })\right)}^{-1/\alpha }$ again. Thus, to then achieve an overall RMSE of *ε*, the first term of $e{\left({\hat{T}}_{M}^{\mathrm{ML}}\right)}^{2}$ is also bounded by *ε*^2^/2. The computational cost of the MLMC estimator is [2]:

3.11 $$C({\hat{T}}_{M}^{\mathrm{ML}})=\sum_{\ell =0}^{L}N_{\ell }\;C_{\ell },$$

where $C_{\ell }:=C(Y_{\ell }^{\left(i\right)})$ represents the cost of a single sample of *Y*_ℓ_.

The variance of the MLMC estimator can be minimized [2] for a fixed computational cost by choosing

3.12 $$N_{\ell }≃\sqrt{\frac{V[Y_{\ell }]}{C_{\ell }}},$$

with the constant of proportionality chosen so that the overall variance is *ε*^2^/2. So, the total cost on level ℓ is proportional to $\sqrt{V[Y_{\ell }]C_{\ell }}$ and hence we can write [31]:

3.13 $$C({\hat{T}}_{M}^{\mathrm{ML}})=\varepsilon^{-2}{\left(\sum_{\ell =0}^{L}\sqrt{V[Y_{\ell }]C_{\ell }}\right)}^{2}.$$

In practice, optimal values for *L* and ${\left\{N_{\ell }\right\}}_{\ell =0}^{L}$ can be computed from sample averages and the unbiased sample variances of *Y*_ℓ_. If we assume that $|E[T_{M}-T]|≃M^{-\alpha }$, then it follows that $|E[Y_{\ell }]|≃M^{-\alpha }$ and $|E[{\hat{Y}}_{L}]|≃M^{-\alpha }$ for *N*_*L*_ sufficiently large, providing us with a computable error estimator to determine either whether *M* is sufficiently large or whether the number of levels *L* needs to be increased.

The above conditions and statements are formally presented in the following theorem [2]:

**Theorem 3.1.**

*Let* ${\hat{Y}}_{\ell }:={\hat{Y}}_{\ell ,N_{\ell }}^{\mathrm{MC}}$ *and suppose that there are positive constants α,β,γ,C*_*α*_*,C*_*β*_*,C*_*γ*_*>0 such that* $\alpha \geq \frac{1}{2}\min(\beta ,\gamma )$ *and*

(i) $|E[T_{M_{\ell }}-T]|\leq C_{\alpha }M_{\ell }^{-\alpha }$*;*
(ii) $V[Y_{\ell }]\leq C_{\beta }M_{\ell }^{-\beta }$*;*
(iii) $C_{\ell }\leq C_{\gamma }M_{\ell }^{\gamma }$.

*Then, for any ε<e*^−1^*, there exist a positive constant C*^*ML*^*, a value L (and corresponding M≡M*_*L*_*) and a sequence* ${\left\{N_{\ell }\right\}}_{\ell =0}^{L}$ *such that
*

$$e{\left({\hat{T}}_{M}^{\mathrm{ML}}\right)}^{2}:=E[{\left({\hat{T}}_{M}^{\mathrm{ML}}-E[T]\right)}^{2}]<\varepsilon^{2}$$

*and
*

$$C({\hat{T}}_{M}^{\mathrm{ML}})=\left\{ \begin{array}{ll} C^{\mathrm{ML}}\varepsilon^{-2}, & \mathrm{if}\;\beta >\gamma ,\\ C^{\mathrm{ML}}\varepsilon^{-2}{\left(\log\varepsilon \right)}^{2}, & \mathrm{if}\;\beta =\gamma ,\\ C^{\mathrm{ML}}\varepsilon^{-2-(\gamma -\beta )/\alpha }, & \mathrm{if}\;\beta <\gamma , \end{array}\right.$$

*whereas
*

$$C({\hat{T}}_{M}^{\mathrm{MC}})=C^{\mathrm{MC}}e^{-2-\gamma /\alpha }$$

*for some positive constant C*^*MC*^.

**Proof.**

The proof is given in [2]. ▪

The MLMC algorithm can be implemented in practice as follows:

(i) Start at the coarsest level (*L*=0).
(ii) Estimate $V[Y_{L}]$ by the sample variance of an initial number of *N*_*L*_ samples.
  Remember that *Y*_0_:=*T*_*M*_0__, i.e. QoI in level 0 (coarsest level) and *Y*_ℓ_:=*T*_*M*_ℓ__−*T*_*M*_ℓ−1__.
(iii) Calculate the optimal *N*_ℓ_,ℓ=0,…,*L*, using (3.12), Remember that $C_{\ell }:=C(Y_{\ell }^{\left(i\right)})$ represents the cost of a single sample of *Y*_ℓ_.
  This step aims to make the variance of the MLMC estimator (3.9) less than $\frac{1}{2}\varepsilon^{2}.$
(iv) Evaluate extra samples at each level as needed for the new *N*_ℓ_.
(v) If *L*≥1, test for convergence using ${\hat{Y}}_{L}≃M^{-\alpha }$.
  Remember that ${\hat{Y}}_{\ell }={\hat{Y}}_{\ell ,N_{\ell }}^{\mathrm{MC}}:=\frac{1}{N_{\ell }}\sum_{i=1}^{N_{\ell }}(T_{M_{\ell }}^{\left(i\right)}-T_{M_{\ell -1}}^{\left(i\right)}).$
  This step tries to ensure that the remaining bias $\left(E[T_{M}-T]\right)$ is less than $(1/\sqrt{2})\varepsilon .$
(vi) If not converged, set *L*=*L*+1 and go back to 2.

The parameters, *α*, *β* and *γ* that can be estimated empirically as follows:

For *γ*, we assume that the number of operations to compute one sample on level ℓ is $C_{\ell }=cM_{\ell }^{\gamma }$ for some constant *c* independent of ℓ. For *β*, we can use as an approximation the slope of the line for log $V[Y_{\ell }]$, m_*β*_, because $V[Y_{\ell }]≃M_{\ell }^{-m_{\beta }}$. For *α*, we can use as an approximation the slope of the line for log $|E[T_{\ell }-T_{\ell -1}]|$, m_*α*_, because $E[T_{\ell }-T_{\ell -1}]≃M_{\ell }^{-m_{\alpha }}$.

### 3.3. Quasi-Monte Carlo simulation

Many of the existing variance reduction methods built upon pseudo-random sequences, e.g. MLMC, are focused on reducing the overall computational cost of a numerical simulation. QMC methods aim to accelerate the rate of convergence of MC by using deterministic (also called quasi-random or low-discrepancy) sequences instead of pseudo-random. The discrepancy of a sequence is a measure of its uniformity and it is computed by comparing the actual number of sample points in a given volume of multidimensional space with the number of sample points that should be there assuming a uniform distribution. These methods normally achieve a convergence rate of $O({\left(\log N\right)}^{M}/N)$. Hence, the convergence rate is directly related to the dimension of the space. This dependence on the dimension of the space together with the correlation of the points generated by the QMC method yields sometimes non-accurate and biased results. That is the main reason why, during the past two decades, QMC methods have been mostly applied to models defined over low-dimensional probability spaces [8,32,33]. In recent years, there has been an increasing interest in tackling the problem of UQ in models of physical processes, for instance, transport in porous media or carbon capture and storage in deep saline aquifers. As discussed in §2, the uncertainty in those models is often represented by truncated KL expansions of log-Gaussian random fields defined in high-dimensional probability spaces. The truncation of these KL expansions adds more uncertainty to the model and this affects the accuracy of the results. Although QMC methods have already been successfully applied to problems defined in high-dimensional spaces by employing different representations of the random inputs [34–37], to the best of our knowledge they have not yet been used in models represented by direct KL decompositions. In this section, we apply the QMC method to an extremely high-dimensional problem with log-Gaussian distributed inputs and present numerical evidence of the acceleration of the MC rate of convergence.

Before introducing the QMC simulation method, let us describe in more detail the MC integration procedure. The MC method uses pseudo-random number sampling algorithms, i.e. during the generation process, uniformly distributed pseudo-random numbers are generated and transformed into the KL coefficients which jointly form random input vectors in $R^{M}$, and these are distributed according to a certain probability distribution, in our case, N(0,I). Let us see an illustrative example: let (Ω,F,P) be a probability space and let $g:{\left[0,1\right]}^{M}\to R$, and *Y* =*g*(*Z*), where *Z* is an uniformly distributed random vector in [0,1]^*M*^. Suppose that we wish to compute $I=\int_{{\left[0,1\right]}^{M}}g(\xi )\;d\xi$ with the MC method. Let *p* denote the uniform probability density function and letting ***ξ*** be uniformly distributed in [0,1]^*M*^, we can apply MC quadrature to approximate *I*, for a given N∈N, in the following way:

$$I=\int_{{\left[0,1\right]}^{M}}g(\xi )d\xi =\int_{{\left[0,1\right]}^{M}}g(\xi )p(\xi )\;d\xi =E[g(\xi )]≃\frac{1}{N}\sum_{j=1}^{N}g(\xi (\omega_{j}))=I_{N},$$

where *ω*_*j*_∈*Ω* and the values $\xi (\omega_{j})\in R^{M}$ are i.i.d. random vectors sampled uniformly by sampling the components *ξ*_*i*_(*ω*_*j*_) independently and uniformly on the interval [0,1].

**Figure 3.**
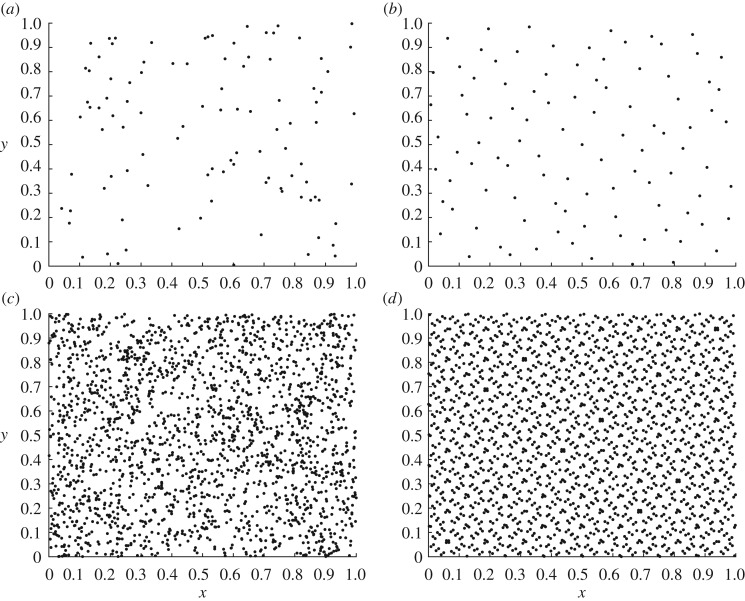
Various pseudo-random and Sobol sequences sampling over the unit square. (*a*) 100 and (*c*) 2000 two-dimensional pseudo-random numbers generated uniformly over the unit square. (*b*) 100 and (*d*) 2000 two-dimensional numbers generated by Sobol sequences over the unit square.

Some examples of quasi-random sequences are: digital nets [38], rank-1 lattice rule [39], Faure sequences [40] or Sobol sequences [41]. From a deep review of the literature, Sobol sequences seem to be the most popular for being used by the QMC method in mathematical models with random inputs [4,6,8], and thus, we will opt for Sobol sequences in this paper. The biggest difference to pseudo-random sequences is that the sample values are chosen under consideration of the previously sampled points, thus avoiding the occurrence of spatial clusters and gaps, as we can observe in figure 3. Figure 3*a* shows 100 pseudo-random numbers sampled from a uniform distribution in the unit square. Figure 3*b* shows the same number of points generated by using a Sobol sequence. It can be observed that the sampling space is filled in a more uniform manner in figure 3*b*. Figure 3*c*,*d* show, respectively, the spatial distribution of 2000 points with pseudo-randon numbers generation and Sobol sequences.

In practice, to implement the QMC method, we use a Sobol sequence to generate *N* points in [0,1]^*M*^. Each of the *M* components of these points can be considered as possible values of the cumulative distribution function of a normally distributed random variable in R. Each of the *N* points are pushed component-wise through the inverse cumulative distribution function of *M* random variables distributed according to N(0,1), to jointly form ${\left\{\xi_{1}^{\left(i\right)},\ldots ,\xi_{M}^{\left(i\right)}\right\}}_{i=1}^{N}$ which are then used as the KL coefficients, and for each of them compute the corresponding travel time $T_{M}^{\left(i\right)}$ for *i*=1,…,*N*.

The QMC estimator used for estimating $E(T_{M})$ in this case is defined as

3.14 $${\hat{T}}_{M,N}^{\mathrm{QMC}}:=\frac{1}{N}\sum_{i=1}^{N}T_{Q,M}^{\left(i\right)},$$

where $T_{Q,M}^{\left(i\right)}$ is the *i*th sample of *T*_*M*_ generated from QMC inputs, and *N* samples are computed in total.

### 3.4. Multilevel Quasi-Monte Carlo simulation

Although there are currently many researchers using MLQMC, there are still very limited works (most of them still *in press*) in the literature [39,42,43]. Thus, to the best of our knowledge, the application of MLQMC to the case of direct KL decompositions for log-Gaussian random fields is also new. This method is a consequence of combining the MLMC algorithm with a randomized QMC estimator instead of the MC estimator. In this paper, we use the MLQMC algorithm developed by Giles & Waterhouse [5]. In order to obtain unbiased estimators for the variances, we need to induce some randomness to the QMC points, this process is known as QMC randomization. There are several ways of QMC randomization, depending on the type of low-discrepancy sequence used. In this study, we use the digital scrambling technique described in [44]. This consists in building a set of *n* (we will use *n*=16 in this study) scrambled Sobol’ sequences to obtain averages for the quantity ${\hat{Y}}_{\ell }$ at level ℓ, i.e. ${\hat{Y}}_{\ell }$ is the average of the quantities ${\hat{T}}_{0}$ and ${\hat{T}}_{\ell }-{\hat{T}}_{\ell -1}$ (for ℓ>0) over the 16 sets of *N*_ℓ_ QMC points. The MLQMC algorithm (described in [5]) can then be summarized as follows:

(i) Start *L*=0.
(ii) Estimate $V[Y_{L}]$ using the 16 sets of QMC points and *N*_*L*_=1.
(iii) While $\sum_{\ell =0}^{L}V[Y_{\ell }]>\varepsilon /2$, double *N*_ℓ_ on the level with largest $V[Y_{\ell }]/(2^{\ell }N_{\ell })$.
(iv) If *L*<2 or the bias estimate is greater than $\varepsilon /\sqrt{2}$, set *L*:=*L*+1 and go to step (ii).

In the following section, numerical results from the application of the above methods to the model problem described in §2 for several discretizations of the physical domain are discussed.

## 4. Numerical results

The procedure followed for conducting the experiments is as follows: firstly, we check (empirically) from which level (i.e. the value of *M*_0_) the asymptotic hypotheses of theorem 3.1 are satisfied (this assures that the simulations at the coarsest grid are reliable approximations of the QoI); secondly, we set the tolerance (MSE) for which we wish the MC algorithms to stop; thirdly, we use the conclusions of theorem 3.1 to implement the four methods as discussed earlier in §3; and finally, the performance of each of the methods is tested by comparing their computational *ε*^2^-Cost, i.e. the number of floating point operations that are needed to achieve the given MSE.

The three tolerances employed for all the comparisons are: 0.01, 0.0064 and 0.0025. The average travel times estimated with MC, MLMC, QMC and MLQMC methods will be denoted, respectively, by *T*_MC_, *T*_MLMC_, *T*_QMC_ and *T*_MLQMC_. The sequence of levels will start with *M*_0_=81. This enables one to get a minimal level of resolution of the problem [2,3]. The maximum level considered will be *M*_5_=66 049 grid points. The other intermediate levels are *M*_1_=289, *M*_2_=1089, *M*_3_=4225 and *M*_4_=16 641.

The conditions of theorem 3.1 for the mean and the variance of the MLMC and MLQMC estimators will be numerically confirmed for each of the cases. The estimates of the parameters *α* and *β* will be computed ‘on the fly’ from sample averages. The dominant cost will rely on the PDE solution, and an algebraic multi-grid method is used as the iterative linear solver. Hence, *γ*=1 in all the simulations. To quantify the cost of the algorithms, we will assume that the number of operations to compute one sample on level ℓ is *C*_ℓ_=*cM*_ℓ_ for some fixed constant *c*, and thus, in the results presented in this paper, we will show the standardized costs, scaled by 1/*c*, i.e. the cost is defined as $\sum_{\ell =0}^{L}N_{\ell }M_{\ell }$.

### 4.1. Comparison between classical Monte Carlo and multilevel Monte Carlo

In this section, we compare the performance of MC and MLMC methods for the tolerances above. As could be expected from similar works in the field and after reviewing the theory related to both methods, the MLMC method clearly outperforms the standard MC. The MLMC results are presented in tables 1–3. The MC results are given in table 4. Thus, by looking at tables 1–4, we observe that while the MLMC method reduces the computational cost of MC for the same degree of accuracy at a rate of 20–26 for tolerances of MSE=0.01 and MSE=0.0025, the reduction reaches its peak at the rate of 80 for a tolerance of MSE=0.0064. Henceforth, in this application, MLMC performs best for a grid of *M*_4_=16 641 elements.

**Table 1.** MLMC estimation with bounds of the average travel time according to a given MSE=0.01. The last row of the first column shows the level at which the code stops.

level ℓ	no. samples, *N*_ℓ_	***ε*^2^**-*Cost* (*ε*^2^=0.01)	*T*_MLMC_	MLMC bounds
0	704	—	—	—
1	93	—	—	—
2	19	—	—	—
3	9	86 784	1.3520	(1.2520, 1.4520)

**Table 2.** MLMC estimation with bounds of the average travel time according to a given MSE=0.0064. The last row of the first column shows the level at which the code stops.

level ℓ	no. samples, *N*_ℓ_	***ε*^2^**-*Cost* (*ε*^2^=0.0064)	*T*_MLMC_	MLMC bounds
0	1103	—	—	—
1	147	—	—	—
2	26	—	—	—
3	12	—	—	—
4	6	222 223	1.3615	(1.2815, 1.4415)

**Table 3.** MLMC estimation with bounds of the average travel time according to a given MSE=0.0025. The last row of the first column shows the level at which the code stops.

level ℓ	no. samples, *N*_ℓ_	***ε*^2^**-*Cost* (*ε*^2^=0.0025)	**T**_MLMC_	MLMC bounds
0	3458	—	—	—
1	613	—	—	—
2	104	—	—	—
3	27	—	—	—
4	10	—	—	—
5	5	908 226	1.3696	(1.3196, 1.4196)

**Table 4.** MC and QMC *ε*^2^-Cost, obtained from the MLMC and MLQMC simulations, respectively, according to the given MSE.

level ℓ	MSE (*ε*^2^)	***ε*^2^**-*Cost***MC**	***ε*^2^**-*Cost***QMC**
3	0.01	1 746 850	582 980
4	0.0064	18 090 227	1 907 358
5	0.0025	23 650 623	13 299 654

Tables 1–3 show the number of samples, *N*_ℓ_, used by the MLMC method in each level, ℓ, for the given MSE, *ε*^2^, the final computational *ε*^2^-Cost (cost for that given tolerance *ε*^2^), the value of the average travel time, *T*_MLMC_, and the corresponding bounds for the estimation (*T*_MLMC_−*ε*,*T*_MLMC_+*ε*).

**Figure 4.**
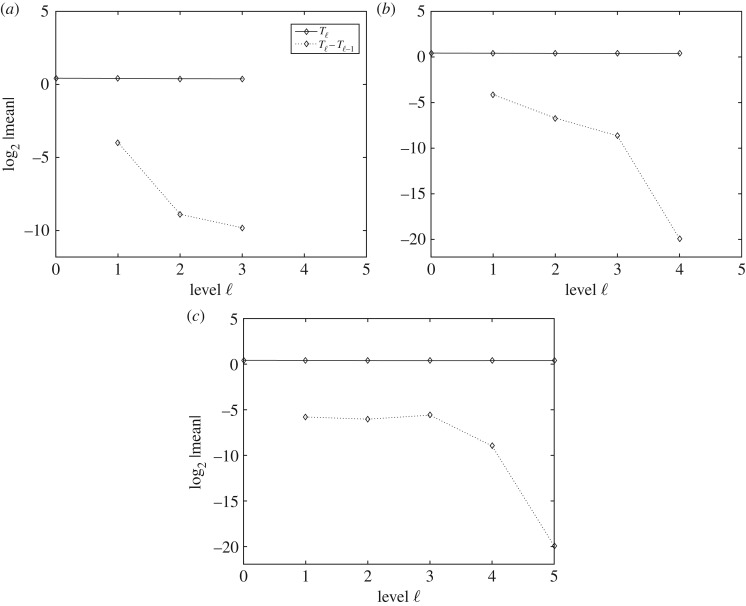
Performance plots for the expectation in the MLMC method. The plots show the numerical verification of the asymptotic behaviour of the expectation of *T* and the convergence of $E[Y_{\ell }]$. Expected values (*a*–*c*) of *T*_ℓ_ and *Y*_ℓ_=*T*_ℓ_−*T*_ℓ−1_, respectively, for MSE=0.01, MSE=0.0064 and MSE=0.0025.

Figure 4 shows the expected value of *T*_ℓ_ and *Y*_ℓ_=*T*_ℓ_−*T*_ℓ−1_ and how the slope of the line for $E[T_{\ell }-T_{\ell -1}]$ has a decreasing tendency. It also shows how $E[T_{\ell }]$ is approximately constant on all levels, numerically verifying condiction (i) of theorem 3.1.

**Figure 5.**
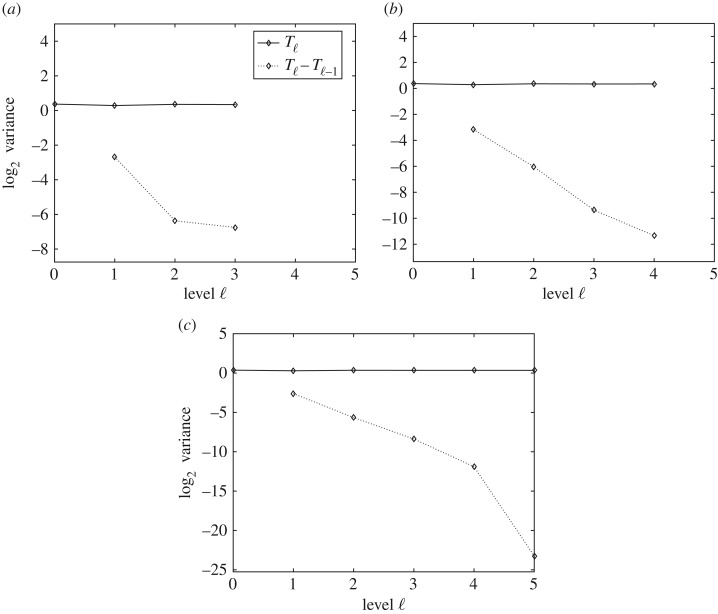
Performance plots for the variance in the MLMC method. The plots show the numerical verification of the asymptotic behaviour of the variance of *T* and the convergence of $V[Y_{\ell }]$. Variances (*a*–*c*) of *T*_ℓ_ and *Y*_ℓ_=*T*_ℓ_−*T*_ℓ−1_, respectively, for MSE=0.01, MSE=0.0064 and MSE=0.0025.

Figure 5 shows the behaviour of the variance of *T*_ℓ_ and *Y*_ℓ_=*T*_ℓ_−*T*_ℓ−1_ for each level ℓ, and how the condition (ii) of theorem 3.1 is numerically satisfied on the levels shown.

### 4.2. Comparison between Monte Carlo and quasi-Monte Carlo

In this section, we compare the performance of MC and QMC methods for the same tolerances used in the previous sections. In this case, low-discrepancy sequences clearly outperform pseudo-random for all the tolerances. Similarly to what happened with MLMC, the reduction in cost provided by the QMC method when compared to MC reaches its peak at level 4. The reduction rate achieved at this level is 9. This could indicate that after the discretization error has been adequately reduced, and consequently, a fine resolution of the QoI is being obtained in each simulation, there is not much additional gain by reducing the sample variance (or sampling error). The latter can be also deduced from figure 9, where after level 4 (or tolerance 0.0064) the slope of the cost for standard MC is nearly constant.

Table 4 provides the data comparison of the computational *ε*^2^-Cost for the MC and QMC. These quantities are obtained in the corresponding MLMC and MLQMC simulations. To calculate the costs for the MC and QMC methods, we use the estimator provided in [3]:

4.1 $$C^{*}=\sum_{\ell =0}^{L}N_{\ell }^{*}M_{\ell },$$

where $N_{\ell }^{*}=2\varepsilon^{-2}V[T_{\ell }]$, so that the variance of the MC (3.3) and QMC (3.14) estimators is $\frac{1}{2}\varepsilon^{2}$ as with the corresponding MLMC and MLQMC methods.

**Figure 6.**
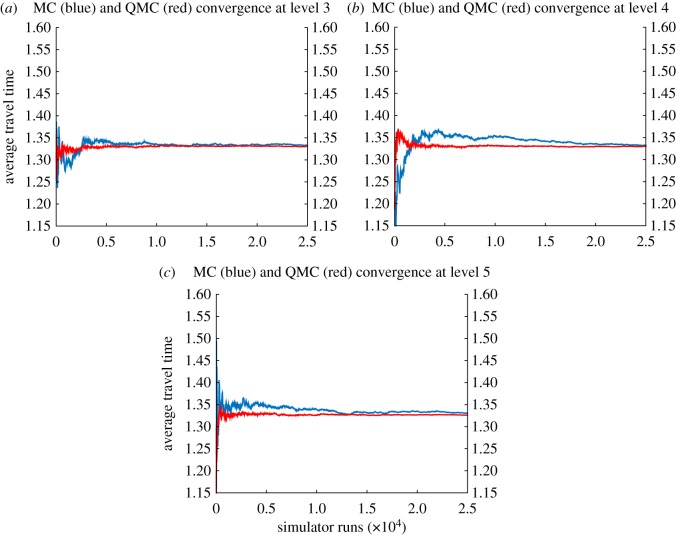
(*a*–*c*) Analysis of the convergence of the MC (blue) and QMC (red) methods for the average travel time at levels 3, 4 and 5. The convergence is calculated over a sample of 25 000 travel times.

In addition to this *ε*^2^-Cost comparison, we will analyse the convergence of the MC and QMC methods at each of the levels where the multilevel methods converged. Figure 6 shows the convergence analysis, based on *N*=25 000 travel times, of MC and QMC methods for the average travel time at levels 3, 4 and 5. Table 5 gives the values of the MC and QMC estimators at each level based on *N*=25 000.

**Table 5.** Comparison of the travel time estimations obtained with the MC and QMC methods at each level.

level ℓ	*T*_MC 25 000 samples	*T*_QMC 25 000 samples
3	1.3255	1.3305
4	1.3312	1.3299
5	1.3253	1.3262

### 4.3. Comparison between quasi-Monte Carlo and multilevel quasi-Monte Carlo

In this section, we compare the performance of QMC and MLQMC methods for the same MSEs as above. In this case, unlike in the comparison between MC and MLMC, MLQMC outperforms QMC in a monotonic order, i.e. the reduction in the cost follows an increasing rate along with the increase in the degree of accuracy (or reduction in tolerance). That is, the reduction rates of MLQMC with respect to QMC are, respectively, 8, 12 and 18 for the tolerances 0.01,0.0064 and 0.0025. These results are within the logic of deterministic sequences generation, and they seem to be (as one could expect) a direct consequence of the ordered (deterministic) way in which the MLQMC estimator is built.

We illustrate next the same tables and figures shown in the previous section for the MC and MLMC methods. Tables 6–8 give the number of samples, *N*_ℓ_, used by the MLQMC method in each level, ℓ, for the given MSE, *ε*^2^, the final computational *ε*^2^-Cost incurred by using the given tolerance, the value of the average travel time, *T*_MLQMC_, and the corresponding bounds for the estimation, (*T*_MLQMC_ − *ε*,*T*_MLQMC_ + *ε*).

**Table 6.** MLQMC estimation with bounds of the average travel time according to a given MSE=0.01. The last row of the first column shows the level at which the code stops.

level ℓ	no. samples, *N*_ℓ_	***ε***^2^-*Cost* (*ε*=0.01)	**T**_MLQMC_	MLQMC bounds
0	488	—	—	—
1	60	—	—	—
2	11	—	—	—
3	10	71 912	1.2985	(1.1985, 1.3985)

**Table 7.** MLQMC estimation with bounds of the average travel time according to a given MSE=0.0064. The last row of the first column shows the level at which the code stops.

level ℓ	no. samples, *N*_ℓ_	***ε*^2^**-*Cost* (*ε*=0.005)	*T*_MLQMC_	MLQMC bounds
0	824	—	—	—
1	109	—	—	—
2	11	—	—	—
3	9	—	—	—
4	4	149 368	1.3427	(1.2627, 1.4227)

**Table 8.** MLQMC estimation with bounds of the average travel time according to a given MSE=0.0025. The last row of the first column shows the level at which the code stops.

level ℓ	no. samples, *N*_ℓ_	***ε*^2^**-*Cost* (*ε*=0.0025)	*T*_MLQMC_	MLQMC bounds
0	2740	—	—	—
1	389	—	—	—
2	57	—	—	—
3	10	—	—	—
4	10	—	—	—
5	5	718 068	1.3550	(1.3005, 1.4050)

**Figure 7.**
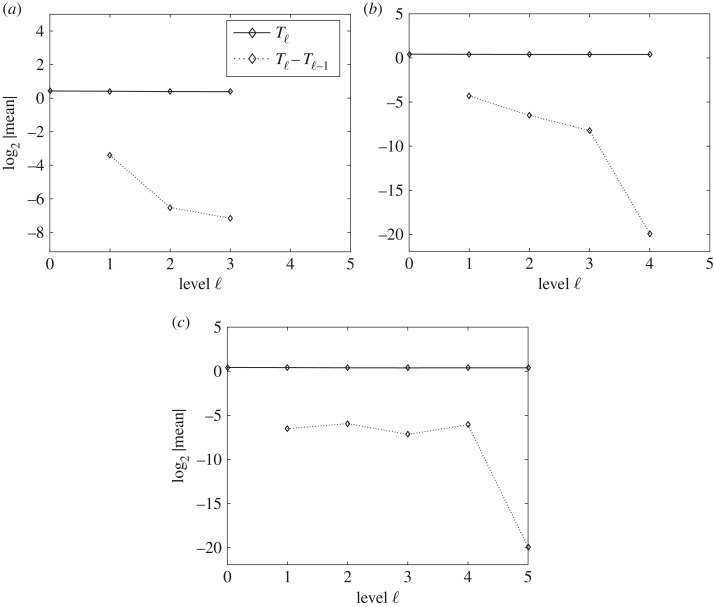
Performance plots for the expectation in the MLQMC method. The plots show the numerical verification of the asymptotic behaviour of the expectation of *T* and the convergence of $E[Y_{\ell }]$. Expected values (*a*–*c*), of *T*_ℓ_ and *Y*_ℓ_=*T*_ℓ_−*T*_ℓ−1_, respectively, for MSE=0.01, MSE=0.0064 and MSE=0.0025.

Figure 7 shows the expected value of *T*_ℓ_ and *Y*_ℓ_=*T*_ℓ_−*T*_ℓ−1_ and how the slope of the line for $E[T_{\ell }-T_{\ell -1}]$ has a decreasing tendency. It also shows how $E[T_{\ell }]$ is approximately constant on all levels.

**Figure 8.**
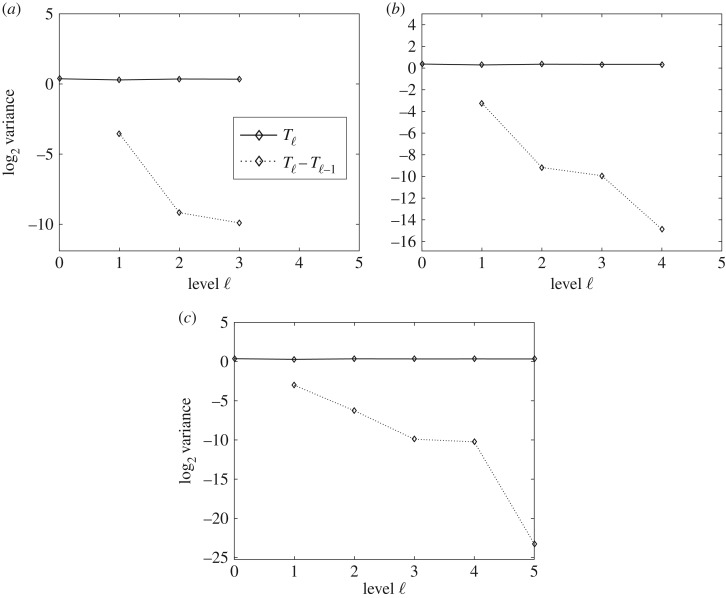
Performance plots for the variance in the MLQMC method. The plots show the numerical verification of the asymptotic behaviour of the variance of *T* and the convergence of $V[Y_{\ell }]$. Variances (*a*–*c*), of *T*_ℓ_ and *Y*_ℓ_=*T*_ℓ_−*T*_ℓ−1_ respectively, for MSE=0.01, MSE=0.0064 and MSE=0.0025.

Figure 8 shows the behaviour of the variance of *T*_ℓ_ and *Y*_ℓ_=*T*_ℓ_−*T*_ℓ−1_ for each level ℓ, and how the condition (ii) of theorem 3.1 is numerically satisfied on the levels shown.

### 4.4. Comparison of Monte Carlo, quasi-Monte Carlo, multilevel Monte Carlo and multilevel quasi-Monte Carlo

**Figure 9.**
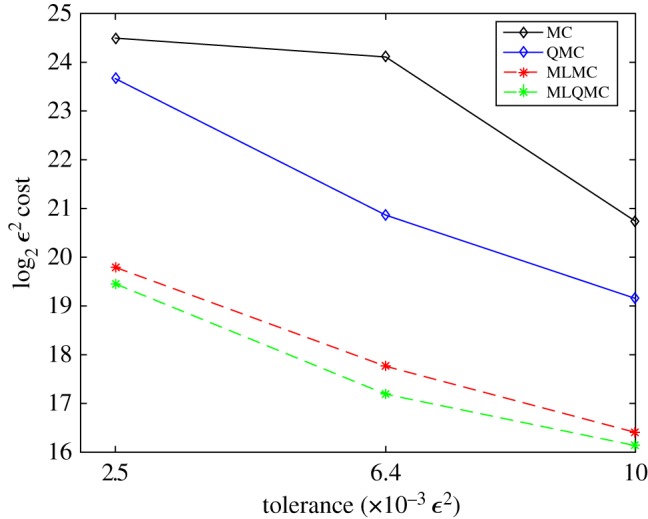
*ε*^2^-Cost for the MC, QMC, MLMC and MLQMC methods for MSE: 0.01, 0.0064 and 0.0025.

The overall picture with the performance of all the methods is shown in figure 9. We can see how the MLQMC method produces a lower computational cost for all the tolerances. MLMC is performing better than MC and QMC, and in conclusion, MC seems to be the least efficient method.

## 5. Conclusion and further work

In this paper, we analysed the efficiency of MC, MLMC, QMC and MLQMC in achieving a desired error level on the estimation of the average travel time during the transport of particles in heterogeneous porous media. The analysis was focused on employing the four methods to solve, under the same conditions, a stochastic model defined in a high-dimensional probability space, and in comparing the computational costs incurred by the four different approaches. The improvements were related to the use of low-discrepancy (Sobol) sequences for the space filling design (QMC) and variance reduction in the multi-grid schemes (MLMC).

One conclusion that can be drawn from the review of the literature and the results obtained in this paper is that, on one hand, for ‘smooth’ uncertain model parameters defined in high dimensions, e.g. the log-Gaussian representation of the hydraulic conductivity in Darcy’s Law, we can rely on QMC methods to significantly reduce the computational cost in an uncertainty analysis, while providing accurate results when compared with other methods like MC. On the other hand, in cases where the uncertain parameters are not smooth enough (e.g. with discontinuities), the QMC method reviewed in this paper may yield inaccurate and biased results. In this case, the use of unbiased randomized QMC estimators as the one used in the MLQMC method might be an alternative, although this would lead to a loss of the deterministic control offered by the standard QMC. A description of such randomized QMC methods is provided in [5].

We provided a detailed comparison of the accuracy and efficiency between the different methods. From the numerical results obtained in the model problem studied in this paper, the QMC and MLMC methods provided the same order of accuracy that the classical MC with considerably less computational runs. The combination of both methods led to the MLQMC method, which was proved to provide the optimal computational effort for the simulator while retaining the same accuracy in the calculations.

In terms of practicality, the multilevel schemes require additional work on the simulator’s numerical code in order to carry out the corresponding multi-grid approach, and this could be impractical for users of Engineering commercial packages for instance. Although the multilevel approaches could also be used for non-nested grids, for non-uniform shapes of the computational domain, methods like the multi-index Monte Carlo [45] could be a better choice.

Further research may include testing the performance of the methods by considering alternative pseudo-random sequences to Sobol when building the QMC and MLQMC estimators, for instance, rank-1 lattice rule [39] or Faure sequences [40]. Refining the MLQMC method discussed in this paper, and therefore reducing its computational cost, is also possible by exploiting the deterministic way in which the estimation of the QoI is conducted, i.e. we could design an algorithm that returns the minimum number of samples needed at each level that makes the statistical error be lower than the given tolerance, instead of using just an exceeding estimation.

## Data accessibility

Our data are deposited at: http://dx.doi.org/10.5061/dryad.ft1d5 [46].
